# Supplementary material for: Retaliatory killing negatively affects African lion (Panthera leo) male coalitions in the Tarangire-Manyara Ecosystem, Tanzania
Source: PLoS One. 2022 Aug 31;17(8):e0272272. doi: 10.1371/journal.pone.0272272 (PMC9432698; doi:10.1371/journal.pone.0272272)
Supplement: S1 Fig — (DOCX) [file pone.0272272.s001.docx]

**“Retaliatory killing negatively affects African lion (Panthera leo) male coalitions in the Tarangire-Manyara Ecosystem, Tanzania”**

**S1 Fig. Matrix of Spearman rank correlation coefficient for predictor variables.** Matrix showing pairwise Spearman rank correlation coefficient values for considered predictor variables. Based on the data collected from March to May 2019 in villages surrounding Tarangire Manyara Ecosystem. Color intensity and the size of the circle are proportional to the correlation coefficients [55]. Correlation coefficient values of less than 0.5 are not correlated and were used as independent variable in our model. Categories are shown in (Table 1). Positive correlations are shown in blue, negative ones in red.
