# Supplementary material for: Retaliatory killing negatively affects African lion (Panthera leo) male coalitions in the Tarangire-Manyara Ecosystem, Tanzania
Source: PLoS One. 2022 Aug 31;17(8):e0272272. doi: 10.1371/journal.pone.0272272 (PMC9432698; doi:10.1371/journal.pone.0272272)
Supplement: S1 Table — (DOCX) [file pone.0272272.s002.docx]

**“Retaliatory killing negatively affects African lion (Panthera leo) male coalitions in the Tarangire-Manyara Ecosystem, Tanzania”**

**S 1 Table. Villages found in Tarangire Manyara Ecosystem.** From 2004 to 2018 Tarangire Lion Project recorded data of lion retaliation incidences occurred in the villages. Selected villages for our survey are marked by an asterix. Villages with a frequency of attacks of ≥ 5 were classified as high retaliation villages (Esilalei, Mswakini chini, Kakoi and Olasiti), those with attack frequencies of ≤ 5 (Mswakini juu, Minjingu, and Oltukai) were classified as low.

| Villages | Frequency | # of lions killed |
| --- | --- | --- |
| Esilalei * | 21 | 27 |
| Loibor Soit | 11 | 22 |
| Olasiti * | 11 | 11 |
| Loibor Siret | 10 | 11 |
| Mswakini chini * | 8 | 7 |
| Tarangire National park | 6 | 6 |
| Kakoi * | 5 | 5 |
| Kimotorok | 5 | 5 |
| Naitolia | 3 | 3 |
| Oltukai * | 3 | 3 |
| Manyara ranch | 4 | 4 |
| Mswakini juu * | 2 | 4 |
| Makuyuni | 2 | 4 |
| Lolkisale | 2 | 5 |
| Emboret | 1 | 1 |
| Minjingu * | 1 | 1 |
| Mbuyuni | 1 | 2 |
| Lemoti | 1 | 4 |
| Engaruka | 1 | 2 |
| Losirwa | 1 | 1 |

Source data: Tarangire Lion Project
