# Supplementary material for: Retaliatory killing negatively affects African lion (Panthera leo) male coalitions in the Tarangire-Manyara Ecosystem, Tanzania
Source: PLoS One. 2022 Aug 31;17(8):e0272272. doi: 10.1371/journal.pone.0272272 (PMC9432698; doi:10.1371/journal.pone.0272272)
Supplement: S2 Table — (DOCX) [file pone.0272272.s003.docx]

***“*Retaliatory killing negatively affects African lion (Panthera leo) male coalitions in the Tarangire-Manyara Ecosystem, Tanzania”**

**S2 Table. Questionnaire to interviewees.** A survey of questions in English that was interviewed to 214 households and 15 key informants from February to May 2019 in the villages surrounding Tarangire Manyara Ecosystem.

| 1. Date (day/month/year) | 2. Survey no | 3.Interviewer (s) name | 4. Other people present at start of interview? (describe) |
| --- | --- | --- | --- |
|  |  |  |  |
| 5. Household GPS | | 6. Village and sub village | |
| S: E: | |  | |

**PART I: SOCIO- DEMOGRAPHIC CHARACTERISTICS OF THE RESPONDENT**

| 7. Occupation (√  Pure Pastoralist  Farmers  Agro-pastoralist  Employees  Business | 8. Age class ( √)  18- 35  36- 45  46-55  Above 55 years | 9.Gender (M/F)  Me=1  Fe=2 | 10. Education level  1 = illiterate,  2 = Primary  3 = Secondary  4 = Tertiary | 11. How long have you been living in TME ( √)  < 5 years  5-15 years  More than 15 years | 12. Benefit from Conservation  1. Employment  2.Business Opportunity  3.Community Development  4. No benefit |
| --- | --- | --- | --- | --- | --- |

**PART 2: AWARENESS AND ATTITUDE ON THE EFFECT OF RETALITORY KILLING OF LIONS**

| 13a. Please rank the wildlife challenges below from the highest to the lowest (1, 2 , 3)  Livestock predation ( ), Crop raiding ( ), Human attacks ( ) |
| --- |
| 13b. Rank the problem animals below 1=most problematic, 2= problematic, 3=least problematic and 4=not problematic  Lion ( ), hyena ( ), leopard ( ), Jackal ( ), elephant ( ), zebra ( ), warthog ( ), wildebeest ( ), tortoise ( ) |
| 13c. From the list above, how often do the problem animals cause problems?  Daily ( ), season dry ( ), season wet ( ), rare ( ) |
| 14. What measures do you use for protecting livestock  Chain-link fence ( ), guard & dogs ( ), tree branches and thorns ( ), having adult herders and not children ( ), having at least two herders ( ), no action ( )  15. How do you rate the of problem animals?  Increasing ( ), Decreasing ( ), Stayed the same ( ) |
| 16. Who is responsible with the problem caused by wildlife ?Government ( ), Community ( ) |
| 17. How do you see the lion population trend in TME  Increasing ( ), decreasing ( ), stayed the same ( ), disappear ( ), I don’t know ( ) |
| 18. Do lions have right to live?  Strongly agree ( ), agree ( ), neutral ( ), disagree ( ), Strongly disagree ( ) |
| 19. Should wildlife be punished when they attack livestock?  Strongly disagree ( ), disagree ( ), neutral ( ), agree ( ), strongly agree ( ) |
| 20. Has retaliation cause a decline in lion population?  Strongly disagree ( ), disagree ( ). Neutral ( ), agree ( ), strongly agree ( ) |
| 21. Has there been a report of lion killing over the following time period?  Within 6 month ( ), every year ( ), every five years ( ), I don’t know ( ), no current record ( ) |

**“Retaliatory killing negatively affects African lion (Panthera leo) male coalitions in the Tarangire-Manyara Ecosystem, Tanzania”**

**S2 Table Dodosa kwa wanakijiji wa TME na watumishi katika taasisi za serikali au zisizo za serikali.**

| 1. Tarehe (siku/mwezi/mwaka) | 2.Dodoso namba: | 3.Jina la anaye hojiwa: | 4.Ididi ya watu wengine waliokuwepo kwenye mahojiano |
| --- | --- | --- | --- |
|  |  |  |  |
| 5. GPS : | | 6. Kijiji na kitongoji: | |
| S: E: | |  | |

**PART I: SIFA ZA KIJAMII ZA WANAOHOJIWA**

| 7. Kazi(√)  Mfugaji tu  Mkulima tu  Mfugaji na mkulima  Mwajiriwa  Mfanyabiashara | 8. Umri/Rika( √)  18- 35  36- 45  46-55  Zaidi ya miaka 55 | 9.Jinsia(Me/Ke)  Me=1  Ke=2 | 10. Kiwango cha Elimu  1 = Hujasoma  2 = Msingi  3 = Sekondari  4 = Elimu ya juu | 11. Umeishi kwa mda gani TME ( √)  < ya miaka 5  5-15  Zaidi ya miaka 15 | 12.Faida kutoka kwa Uhifadhi  1. Ajira  2.Fursa za kufanya biashara  3.Maendeleo katika jamii  4. Hakuna faida |
| --- | --- | --- | --- | --- | --- |

**PART 2: UFAHAMU NA MTAZAMO WA WATU KATIKA ATHARI ZITOKANAZO NA MAUAJI YA SIMBA**

| 13a.Tafadhali ainisha katika mtiririko athari zitokanazo na wanyamapori, kuanza na inayoongoza hadi mwisho  Mauaji ya mifugo ( ), Uvamizi wa mazao ( ), Shambulio kwa mtu/watu ( ) |
| --- |
| 13b. Tafadhali chagua hapa chini wanyama wenye shida kulingana na athari wnazoleta; 1=Msumbufu sana, 2=Msumbufu, 3=Msumbufu kidogo na 4=siyo msumbufu  Simba ( ), Fisi ( ), Chui( ), Bweha ( ), Tembo ( ), Pundamilia ( ), Nguruwe pori ( ), Nyumbu ( ), Kobe ( ) |
| 13c. Ni mara ngapi wanyama tajwa hapo juu wanaleta shida?  Kila siku ( ), Katika kiangazi ( ), Katika masika ( ), mara chache ( ) |
| 14. Njia zipi unatumia katika kulinda/ kutunza mifugo yako?  Uzio wa waya ya chuma ( ), mchungi mtoto na mbwa ( ), masanzu ( ), mchungi kijana ( ), kuwa na wachungi wawili na zaidi( ), sifanyi chochote ( )  15.Shida zinazolotana wanyama tajwa hapo juu je?  Zinaongezeka(), Zinapungua ( ), zipo vile vile ( ) |
| 16. Nani anahusika na matatiizo yanayoletwa na wanyama pori? Serikali ( ), Jami/wnakijiji |
| 17. Unaionaje idadi ya simba katika eneo la ekologia ya Tarangire na manyara?  Inaongezeka ( )inapungua ( ), ipo hivyo hivyo ( ), wamepotea ( ), sijui( ) |
| 18. Je Simba wanahaki ya kuendelea kuwepo?  Nakubaliana sana ( ), kukubali ( ), sijui( ), sikubali, sikubali sana ( ) |
| 19. Adhabu zitolewe kwa wanyama wakali pindi wanapoa vamia mifugo?  Sikubali sana ( ), sikubali( ), sijui ( ), nakubali ( ), nakubali sana ( ) |
| 20. Je mauaji ya Simba yamesababisha inadi yao kupungua?  Sikubali sana ( ), sikubali( ), sijui ( ), nakubali ( ), nakubali sana ( ) |
| 21. Umepata taarifa kuhusu yoyote kuhusu mauaji ya simba katika vipindi vifuatavvyo?  Ndani ya miezi 6 ( ), Kila mwaka ( ), kila miaka mitano ( ), Sijui ( ), sijapata taarifa yoyote ( ) |
