# Supplementary material for: Retaliatory killing negatively affects African lion (Panthera leo) male coalitions in the Tarangire-Manyara Ecosystem, Tanzania
Source: PLoS One. 2022 Aug 31;17(8):e0272272. doi: 10.1371/journal.pone.0272272 (PMC9432698; doi:10.1371/journal.pone.0272272)
Supplement: S3 Table — (DOCX) [file pone.0272272.s004.docx]

**“Retaliatory killing negatively affects African lion (Panthera leo) male coalitions in the Tarangire-Manyara Ecosystem, Tanzania”**

**S3 Table**. **Effect of retaliation on coalition size.** A priori generalized linear mixed models representing the effect of retaliatory killing on coalition size based on data collected in Tarangire Manyara Ecosystem from 2004 to 2018. Description of the variables are as in (Table 1). Explanatory variables were added in candidate models with coalition ID as random effect. df: degrees of freedom; AICc: Akaike’s Information Criterion corrected for small sample size (n = 46); ΔAICc: difference in AICc values between the best performing model and the model of interest; *ω_i_*: Akaike model weights. PA = protected area.

| S/N | Candidate Models | Df | AICc | ΔAICc | *ω_i_* |
| --- | --- | --- | --- | --- | --- |
| 1. | Retaliation risk | 43 | 151.5 | 0.00 | 0.28 |
| 2. | Hunting location | 43 | 151.9 | 0.53 | 0.22 |
| 3. | PA location | 43 | 152.0 | 0.56 | 0.21 |
| 4. | Hunting location, retaliation risk | 42 | 153.7 | 2.32 | 0.09 |
| 5. | PA location, retaliation risk | 42 | 153.8 | 2.40 | 0.08 |
| 6. | PA location, hunting location | 42 | 154.3 | 2.89 | 0.06 |
| 7. | Retaliation risk, hunting location, PA location | 41 | 156.2 | 4.83 | 0.02 |

Commas (,) -Separate independent factors

DF- “degrees of freedom”
