# Supplementary material for: Retaliatory killing negatively affects African lion (Panthera leo) male coalitions in the Tarangire-Manyara Ecosystem, Tanzania
Source: PLoS One. 2022 Aug 31;17(8):e0272272. doi: 10.1371/journal.pone.0272272 (PMC9432698; doi:10.1371/journal.pone.0272272)
Supplement: S4 Table — (DOCX) [file pone.0272272.s005.docx]

**“Retaliatory killing negatively affects African lion (Panthera leo) male coalitions in the Tarangire-Manyara Ecosystem, Tanzania”**

**S4 Table. Effect of retaliation on tenure period of male coalitions**. A priori generalized linear mixed models representing the effect of retaliatory killing on tenure period of male coalitions based on data collected in Tarangire Manyara Ecosystem from 2004 to 2018. Description of the variables are as in (Table 1). Explanatory variables were added in candidate models with coalition ID as random effect; df: degree of freedom; AICc: Akaike’s Information Criterion corrected for small sample size (n=46); ΔAICc: difference in AICc values between the best performing model and the model of interest; *ω_i_*: Akaike model weights.

| S/N | Candidate Models | Df | AICc | ΔAICc | ω_i_ |
| --- | --- | --- | --- | --- | --- |
| 1. | Male numbers, PA location | 42 | 351.0 | 0.00 | 0.21 |
| 2. | PA location | 43 | 351.1 | 0.12 | 0.20 |
| 3. | Male numbers | 43 | 351.3 | 0.38 | 0.17 |
| 4. | Retaliation risk | 43 | 351.8 | 0.84 | 0.14 |
| 5. | Male numbers*retaliation risk, PA location | 40 | 352.8 | 1.84 | 0.08 |
| 6. | Male numbers, PA location, retaliation risk | 41 | 353.0 | 2.09 | 0.07 |
| 7. | Male numbers, PA location, retaliation risk, hunting location | 40 | 355.7 | 4.71 | 0.02 |

Commas (,) - Separates independent factors

Asterisk (*)- Denotes interaction

DF- Degree of Freedom
