# Supplementary material for: Retaliatory killing negatively affects African lion (Panthera leo) male coalitions in the Tarangire-Manyara Ecosystem, Tanzania
Source: PLoS One. 2022 Aug 31;17(8):e0272272. doi: 10.1371/journal.pone.0272272 (PMC9432698; doi:10.1371/journal.pone.0272272)
Supplement: S5 Table — (DOCX) [file pone.0272272.s006.docx]

**“Retaliatory killing negatively affects African lion (Panthera leo) male coalitions in the Tarangire-Manyara Ecosystem, Tanzania”**

**S5 Table. Community attitude towards lions**. A priori generalized linear mixed models representing predictor variables that determine the attitude of the community whether lions have the right to live or not. Data were collected during interviews, n= 214 from March to May 2019 in villages around Tarangire Manyara Ecosystem. Description of the variables are in table 1. Explanatory variables were added in the candidate model with village ranks as random effect, df: degree of freedom; AICc: Akaike’s Information Criterion corrected for small sample size (n=214); ΔAICc: difference in AICc values between the best performing model and the model of interest; *ω_i_*: Akaike model weights.

| S/N | Candidate models | df | AICc | ∆AICc | ωi |
| --- | --- | --- | --- | --- | --- |
| 1. | Age class, education | 4 | 254.2 | 0.00 | 0.18 |
| 2. | Benefit | 3 | 254.3 | 0.11 | 0.17 |
| 3. | Occupation | 3 | 254.8 | 0.54 | 0.14 |
| 4. | Benefit, occupation | 4 | 255.0 | 0.83 | 0.12 |
| 5. | Education, age class, sex | 5 | 256.0 | 1.82 | 0.07 |
| 6. | Age class, sex | 4 | 256.2 | 1.98 | 0.06 |
| 7. | Education, occupation | 4 | 256.2 | 2.03 | 0.06 |
| 8. | Resident | 3 | 256.8 | 2.58 | 0.05 |
| 9. | Education | 5 | 257.1 | 2.9 | 0.04 |
| 10 | Resident, age class, sex | 5 | 258.2 | 3.97 | 0.02 |
| 11 | Education, occupation, resident | 5 | 258.3 | 4.12 | 0.02 |
| 12 | Age class, education, benefit, occupation, sex, resident | 8 | 259.5 | 5.33 | 0.01 |

Commas (,) -Separate independent factors. Variables are in Table 1

**DF- “Degrees of freedom”**
