# Supplementary material for: Retaliatory killing negatively affects African lion (Panthera leo) male coalitions in the Tarangire-Manyara Ecosystem, Tanzania
Source: PLoS One. 2022 Aug 31;17(8):e0272272. doi: 10.1371/journal.pone.0272272 (PMC9432698; doi:10.1371/journal.pone.0272272)
Supplement: S6 Table — (DOCX) [file pone.0272272.s007.docx]

**“Retaliatory killing negatively affects African lion (Panthera leo) male coalitions in the Tarangire-Manyara Ecosystem, Tanzania”**

**S6 Table.** **Attitude of the community whether lions should be killed after livestock depredation.** A priori generalized linear mixed models representing predictor variables on whether lions should be killed based on the frequency of depredation on livestock. Data were collected during interviews, n= 214 from March to May 2019 in villages around Tarangire Manyara Ecosystem. Categories of the variables are in table 1 and explanatory variables added in candidate models with village name as random effect; df: degree of freedom; AICc: Akaike’s Information Criterion corrected for small sample size; ΔAICc: difference in AICc values between the best performing model and the model of interest; ωi: Akaike model weights.

| S/N | Candidate models | df | AICc | ∆AICc | ωi |
| --- | --- | --- | --- | --- | --- |
| 1 | Education | 3 | 293.8 | 0 | 0.29 |
| 2 | Benefit | 3 | 294.1 | 0.27 | 0.25 |
| 3 | Education, occupation | 4 | 295.7 | 1.88 | 0.11 |
| 4 | Benefit, occupation | 4 | 296.0 | 2.15 | 0.1 |
| 5 | Occupation | 3 | 297.1 | 3.25 | 0.05 |
| 6 | Resident | 3 | 297.4 | 3.55 | 0.04 |
| 7 | Education, occupation, resident | 5 | 297.6 | 3.74 | 0.04 |
| 8 | Education, age class, sex | 5 | 297.6 | 3.75 | 0.04 |
| 9 | Age class, sex | 4 | 299.6 | 5.74 | 0.01 |
| 10 | All | 8 | 300.6 | 6.79 | 0 |

Commas (,) -Separate independent factors
