# Supplementary material for: Retaliatory killing negatively affects African lion (Panthera leo) male coalitions in the Tarangire-Manyara Ecosystem, Tanzania
Source: PLoS One. 2022 Aug 31;17(8):e0272272. doi: 10.1371/journal.pone.0272272 (PMC9432698; doi:10.1371/journal.pone.0272272)
Supplement: S7 Table — (DOCX) [file pone.0272272.s008.docx]

**“Retaliatory killing negatively affects African lion (Panthera leo) male coalitions in the Tarangire-Manyara Ecosystem, Tanzania”**

**S7 Table. Male lion coalition groups for ten individual prides**. Summary of data for male lion coalitions based on data collected by Tarangire Lion Project from 2004 to 2018 in Tarangire Manyara Ecosystem. Column contains pride name, Coalition ID column indicate the name of the coalition group found in the pride, i.e ALC1 this is the first coalition in the pride named altipiano, number of males (i.e., the size of the coalition group), tenure period (showing the time period that coalition group lasted in the pride) and location (whether the home range area of the coalition group was located within the park = midst, or in the periphery).

| **Pride name** | **Coalition ID** | **# of males** | **Tenure period (months)** | **Location** |
| --- | --- | --- | --- | --- |
| Altipiano | ALC5 | 2 | 6 | Periphery |
| Altipiano | ALC2 | 3 | 10 | Periphery |
| Altipiano | ALC4 | 2 | 11 | Periphery |
| Altipiano | ALC6 | 2 | 12 | Periphery |
| Altipiano | ALC3 | 2 | 19 | Periphery |
| Altipiano | ALC1 | 2 | 24 | Periphery |
| Altipiano | ALC7 | 2 | 4 | Periphery |
| Boundary hill | BC31 | 3 | 14 | Periphery |
| Kuro | KC37 | 2 | 4 | Midst |
| Kuro | KC32 | 1 | 12 | Midst |
| Kuro | KC34 | 1 | 12 | Midst |
| Kuro | KC39 | 2 | 12 | Midst |
| Kuro | KC38 | 5 | 17 | Midst |
| Kuro | KC35 | 3 | 20 | Periphery |
| Kuro | KC33 | 2 | 22 | Periphery |
| Kuro | KC40 | 3 | 26 | Midst |
| New Silale | NSC42 | 2 | 4 | Periphery |
| New Silale | NSC41 | 2 | 12 | Periphery |
| New Silale | NSC43 | 1 | 12 | Periphery |
| New Tarangire hill | NTC21 | 2 | 25 | Midst |
| New Tarangire hill | NTC20 | 3 | 52 | Midst |
| New wazi | NWC9 | 3 | 8 | Periphery |
| New wazi | NWC12 | 4 | 24 | Midst |
| New wazi | NWC10 | 2 | 25 | Periphery |
| New wazi | NWC11 | 1 | 39 | Periphery |
| Old Silale | OSC46 | 1 | 12 | Periphery |
| Old Silale | OSC45 | 5 | 24 | Periphery |
| Old Silale | OSC44 | 2 | 48 | Periphery |
| Silale/Minynyo | SM47 | 2 | 6 | Midst |
| Silale/Minynyo | SM48 | 1 | 29 | Midst |
| Silale/Minynyo | SM49 | 3 | 44 | Midst |
| Silale/Minynyo | SM50 | 5 | 74 | Midst |
| Tarangire hill | TC23 | 3 | 3 | Periphery |
| Tarangire hill | TC27 | 3 | 4 | Periphery |
| Tarangire hill | TC29 | 3 | 6 | Periphery |
| Tarangire hill | TC28 | 2 | 10 | Periphery |
| Tarangire hill | TC25 | 2 | 12 | Periphery |
| Tarangire hill | TC26 | 2 | 19 | Periphery |
| Tarangire hill | TC30 | 4 | 25 | Periphery |
| Tarangire hill | TC22 | 2 | 35 | Midst |
| Tarangire hill | TC24 | 5 | 55 | Periphery |
| Wazi | WC13 | 3 | 6 | Periphery |
| Wazi | WC15 | 2 | 12 | Periphery |
| Wazi | WC17 | 1 | 12 | Periphery |
| Wazi | WC16 | 2 | 16 | Periphery |
| Wazi | WC14 | 2 | 38 | Periphery |
