# Supplementary material for: Retaliatory killing negatively affects African lion (Panthera leo) male coalitions in the Tarangire-Manyara Ecosystem, Tanzania
Source: PLoS One. 2022 Aug 31;17(8):e0272272. doi: 10.1371/journal.pone.0272272 (PMC9432698; doi:10.1371/journal.pone.0272272)
Supplement: S8 Table — (DOCX) [file pone.0272272.s009.docx]

**“Retaliatory killing negatively affects African lion (Panthera leo) male coalitions in the Tarangire-Manyara Ecosystem, Tanzania”**

**S 8 Table. Summary of the response variable tenure period.** Parameter estimates and 95% confidence intervals (CI) for the top-ranking model for the effect of retaliatory killing on the tenure period of male coalitions. Based on data collected by Tarangire Lion Project from 2004 to 2018 in the Tarangire-Manyara ecosystem.

| Parameter | Estimate | SE | Lower CI | Upper CI | P |
| --- | --- | --- | --- | --- | --- |
| Male numbers | 0.13 | 0.08 | -0.03 | 0.31 | 0.236 |
| PA location | -0.34 | 0.20 | -0.75 | 0.06 | <0.001 |
| Retaliation risk | 0.34 | 0.21 | -0.09 | 0.77 | 0.221 |

PA= Protected area
