# Supplementary material for: Retaliatory killing negatively affects African lion (Panthera leo) male coalitions in the Tarangire-Manyara Ecosystem, Tanzania
Source: PLoS One. 2022 Aug 31;17(8):e0272272. doi: 10.1371/journal.pone.0272272 (PMC9432698; doi:10.1371/journal.pone.0272272)
Supplement: Attachment — Submitted filename: Lion manuscript.pdf [file pone.0272272.s014.pdf]

Report on Lion manuscript No. PONE - D 21 - 29635

1. **introduction**

Line 52: what was the estimated lion population 21 years ago?

Line 60 humans

Line 61 human attack/injuries

Line 61/62 Such losses of life and property

Line 64 ….. persistence of carnivore populations in many areas

Line 80/81: The size of a coalition group is an important component in ensuring the survival of cubs, females and the pride as a whole and can be a good indicator of population fitness

Line 81/82/83: the longer the time a coalition group with three or more individuals can last in different prides, the more they ensure the survival of their offspring

Line 85: how the killing of

Line 118: Our study will contribute in providing information on the effect of retaliation on the social grouping of lions…..

2. **Materials and methods**

Line 128: about 10.0%

Line 185: The questionnaire was tested in a pilot survey using 10 individuals living in TME

Line 186: was in English and was translated in

Line 233: Table 1

Line 234: always use "degrees of freedom"

Line 254: Table 4

Line 255: “degrees of freedom”

Line 265: variables are in Table 4

Line 266: degrees of freedom

3. Results

Line 288: areas with high risk of retaliation and exhibited a longer tenure

Lines 330/331/332: What is researchers account in comparison to the interviewees’ responses?

Line 334: in return for their attacks on livestock

Any gender aspect in human – lion conflicts?

4. Discussion

Lines 394/395: Our findings suggest that younger and higher educated people were more tolerant towards conservation of lions.

Lines 401/402: and use of adults to guide

Line 408: to humans

Lines 412/413/414: In contrast, more livestock attacks by hyenas (*Crocuta crocuta*) and incidences of human injury were reported in enclosures which align with reports from other studies….

Lines 421/422: Non - governmental organizations

Line 454: References

General comment:

The paper has reported on the key facts regarding human – lion conflicts in the study area. The findings have direct bearing and application to the management and conservation of lion populations and the handling of human – lion conflicts. The research design, data collection protocols, data processing and analysis, results and discussion are adequate. There are some minor observations above for addressing.
