# Supplementary material for: Retaliatory killing negatively affects African lion (Panthera leo) male coalitions in the Tarangire-Manyara Ecosystem, Tanzania
Source: PLoS One. 2022 Aug 31;17(8):e0272272. doi: 10.1371/journal.pone.0272272 (PMC9432698; doi:10.1371/journal.pone.0272272)
Supplement: Attachment — Submitted filename: Response to Reviewers.docx [file pone.0272272.s015.docx]

PONE-D-21-29635

Retaliatory killing negatively affects African lion (*Panthera leo*) male coalitions in the Tarangire-Manyara Ecosystem, Tanzania.

PLOS ONE

Dear Academic editor and Reviewers

*We thank you for your support in terms of inputs and comments on the submitted manuscript to your journal.*

*Here below we have addressed issues identified by Academic editor chronologically to how they were outlined. The revised parts are highlighted in yellow in the revised manuscript.*

Journal Requirements:

When submitting your revision, we need you to address these additional requirements.

1. Please ensure that your manuscript meets PLOS ONE's style requirements, including those for file naming. The PLOS ONE style templates can be found at

<https://journals.plos.org/plosone/s/file?id=wjVg/PLOSOne_formatting_sample_main_body.pdf>

*We thank the editor for your support and we have prepared the revised manuscript following the guidelines as per the given link of PLOS ONE format.*

2. Please include additional information regarding the survey or questionnaire used in the study and ensure that you have provided sufficient details that others could replicate the analyses. For instance, if you developed a questionnaire as part of this study and it is not under a copyright more restrictive than CC-BY, please include a copy, in both the original language and English, as Supporting Information. If the original language is written in non-Latin characters, for example Amharic, Chinese, or Korean, please use a file format that ensures these characters are visible.

*We thank the editor for their guidance and have put the copies of the questionnaire (both English and Kiswahili versions) used in this study as Supporting Information titled S2 and S2.1 tables.*

3. We note that Figures 1 in your submission contain map images which may be copyrighted. All PLOS content is published under the Creative Commons Attribution License (CC BY 4.0), which means that the manuscript, images, and Supporting Information files will be freely available online, and any third party is permitted to access, download, copy, distribute, and use these materials in any way, even commercially, with proper attribution. For these reasons, we cannot publish previously copyrighted maps or satellite images created using proprietary data, such as Google software (Google Maps, Street View, and Earth). For more information, see our copyright guidelines: <http://journals.plos.org/plosone/s/licenses-and-copyright>

We require you to either (1) present written permission from the copyright holder to publish these figures specifically under the CC BY 4.0 license, or (2) remove the figures from your submission:

1. You may seek permission from the original copyright holder of Figures 1 to publish the content specifically under the CC BY 4.0 license.

We recommend that you contact the original copyright holder with the Content Permission Form (http://journals.plos.org/plosone/s/file?id=7c09/content-permission-form.pdf) and the following text:

“I request permission for the open-access journal PLOS ONE to publish XXX under the Creative Commons Attribution License (CCAL) CC BY 4.0 (http://creativecommons.org/licenses/by/4.0/). Please be aware that this license allows unrestricted use and distribution, even commercially, by third parties. Please reply and provide explicit written permission to publish XXX under a CC BY license and complete the attached form.”

Please upload the completed Content Permission Form or other proof of granted permissions as an "Other" file with your submission.

In the figure caption of the copyrighted figure, please include the following text: “Reprinted from [ref] under a CC BY license, with permission from [name of publisher], original copyright [original copyright year].”

2. If you are unable to obtain permission from the original copyright holder to publish these figures under the CC BY 4.0 license or if the copyright holder’s requirements are incompatible with the CC BY 4.0 license, please either i) remove the figure or ii) supply a replacement figure that complies with the CC BY 4.0 license. Please check copyright information on all replacement figures and update the figure caption with source information. If applicable, please specify in the figure caption text when a figure is similar but not identical to the original image and is therefore for illustrative purposes only.

The following resources for replacing copyrighted map figures may be helpful:

USGS National Map Viewer (public domain): http://viewer.nationalmap.gov/viewer/

The Gateway to Astronaut Photography of Earth (public domain): http://eol.jsc.nasa.gov/sseop/clickmap/

Maps at the CIA (public domain): https://www.cia.gov/library/publications/the-world-factbook/index.html and https://www.cia.gov/library/publications/cia-maps-publications/index.html

NASA Earth Observatory (public domain): http://earthobservatory.nasa.gov/

Landsat: http://landsat.visibleearth.nasa.gov/

USGS EROS (Earth Resources Observatory and Science (EROS) Center) (public domain): http://eros.usgs.gov/#

Natural Earth (public domain): <http://www.naturalearthdata.com/>

*We thank the editor again for this guidance and have managed to get permission, on the use of the shape files that were used to draw the map of Fig 1 and Fig 2. The source of the shape file(s) used to produce the figures have been cited in the caption of fig 1 found in Line 135 to 138, and fig 2 in Line 233 to 234. Also, we have reduced information on the map of fig 1 to allow easy visualization and improve the map quality.*

Additional Editor Comments:

Overall, this manuscript needs to be reviewed for English language fluency. Throughout the text, some of the phrases and sentences are unclear. I point out several of those areas below, but there are many more.

Importantly, you are missing a lot of information for your reader to be able to follow what you did in your study and what resulted from it. Additionally, superfluous information is added in places making the manuscript more confusing to follow. It might be worth looking at other published manuscripts to understand the clarity of detail that needs to be provided for a submission to PLOS ONE.

*We thank the editor for this useful comment and we have consulted the recent papers from the journal and revised sections of our current manuscript and have addressed each revision listed below in detail.*

*Also, we have removed superfluous information, edited and added lines to clarify sentences in various sections in the revised manuscript as suggested by the reviewers below.*

In the Introduction, the structure is somewhat jumbled. It needs to be re-organized to flow from the general to the more specific, or at least sticking with a clear topic within a paragraph. Currently the text jumps around, and includes information that should be removed, reorganized, or moved to other sections.

*We thank the editor for this comment and have revised the introduction section from line 44 to line 120. We have re-organized the text to flow from general to specific and sticking to clear topic within a paragraph as highlighted in yellow lines of the revised manuscript.*

Some of your citations in the text are misleading in that the authors you cite are not specifically writing about the topics you cite them for. Take care to cite properly.

*We thank the editor for this input. We have read and addressed the matter by cross checking the cited article with the content of the paragraph and following the guideline of journal format. We have replaced the missing in-text citations in lines 56 and 57. All new in-text citations have been referenced according to plos one guidelines in lines 59 and 61.*

In your Results, you provide outputs from your models but not the basic statistics to allow your readers to understand the conclusions you discuss later in the paper. This is a critical publication criterion for PLOS ONE -- your reader needs to be able to follow you through to your conclusions, which currently is not the case. For example, you discuss negative impacts on coalitions, but you do not provide the results to support this.

Further, you do not link your discussion back to all of your model results. If you are going to keep those analyses, use them in your text. If not, remove them. Depending on which results you want to focus on, you might want to reconsider which results go in Supplemental and which belong in the main text.

*We thank the editor for the comment. We have included basic statistics of the results obtained for each response variable in excel sheet supporting information (S8 Text) and (S9 Text). And have linked these results to discussion and later reflected to this to the conclusion section of the revised paper.*

*Thank you for your comment editor. We have also revised the manuscript and managed to link the models of the results, to the discussion. We have linked lines 236 - 238 in the results with lines 308 - 310 and 311 - 313 in the discussion. Results from lines 236 - 238 have been linked with supporting information (S7).*

*Also, results from lines 245 to line 248 have been linked to lines 326 - 329 in the discussion for the response variable of tenure period. And for assessing the attitude of the community on the effect of retaliation to lion population line 272 to line 275 in the results have been linked to line 340 to line 362.*

Your Discussion provides interesting information but does not focus on discussing your results as much as bringing in evidence from other studies. For example, in your Conclusion, you state that retaliatory killing has negative impacts on coalitions but you don’t discuss this idea. It’s good that you are comparing your work with other studies, but you need to first focus on your own results before the reader can follow you in comparing to others’ results.

*We thank the editor for the comments, we have added information that are detailed explaining more the meaning of the results from this study before comparing with findings from other researchers. As seen in the added text bolded in yellow under discussion section.*

In terms of clarity, some of your uses of “/” or “i.e.” within a sentence should be removed. In these instances, think through what message you are trying to convey and write in plain words.

*We appreciate editor remarks, we have made changes on the use of the mentioned words and symbols and also put more effort in the use of more plain words.*

As a side note, it might be worth being aware of this paper,

Romanach, S.S., Lindsey, P.A. and Woodroffe, R., 2007. Determinants of attitudes towards predators in central Kenya and suggestions for increasing tolerance in livestock dominated landscapes. Oryx, 41(2), pp.185-195.

*We thank editor for the suggestion of the paper.*

*For each line mentioned below comments, suggestion and correction advised have been done.*

- Line 55: consider changing “open” to “natural”

*Changes were made from open to natural at line 50 in the revised manuscript*

- L 59: change to, “in most of”

*Changes were made to line 52 was written in most of in the revised manuscript*

- L 68: social grouping of what? Use caution when you write “nothing has been studied” unless you are certain this is the case. Your phrasing starting at the end of L 86 is preferable.

*Changes were made to line 61 from “nothing has been studied” to “little is known on the effect of retaliation to carnivore population”. In line 61- 62 we have omitted the word social grouping and managed to re write the sentence properly.*

- L 75: you state above that prides are females so be clear in the text what the role of males is, and perhaps clarify the relationship between prides and coalitions in L 70

*From line 63 lion prides consist of females and males. Lines 64 to 65, discuss that female in a pride are responsible for giving birth and communal rearing of the offspring to independent age,*

*The relationship of male lions and pride are in line 66- 67, males form coalition to ensure protection, maximum reproduction and hunting success changes are found in the revised manuscript*

- L 90: you need to define what makes an area high vs low risk

*In line 89 and 93 of the revised manuscript, we have added information on the grouping of an area either high or low risk of retaliation has been done based on the record of lion killing in the village areas (S1 Table).*

- L 94: adjust to give the name then the citation number after

*Changes have been made and name of the author has been written (Woodroffe) in line 14 on the list of reference of the revised manuscript*

- L 93-98: tighten up and clarify this section. The idea of hunting comes mixed in with retaliatory killings and will not be clear to the reader that you have now switched to trophy hunting at first mention of hunting.

*The section has been clarified from line 85 to line 88 explaining the effect of hunting to male coalition and lion population. Further line 89 to line 93 has clarified the effect of retaliation to lions. The concept of hunting has been clarified to enable the reader to flow through the text found in the revised manuscript.*

- Additionally, in the same lines above, what you included in your analyses should go in the Methods, not your Introduction

*Line 96 has been taken to the method section line 162 of the revised manuscript*

- L 99: tell your reader what a hunting block is, many will not be familiar with trophy hunting lingo

*Definition of a hunting block has been added in line 85 of the revised manuscript*

- L 113: where were the data collected? TME?

*The lion monitoring data from 2004 to 2018 were collected in Tarangire-Manyara Ecosystem for the identified lion prides that were daily tracked and monitored. This information has been added in line 113 to 115 of the revised manuscript.*

- L 114: it’s not clear what you mean by “coalition groups” in this context. You can give the details in the Methods, but as a general term, it’s not clear if you mean size of the group other metrics.

*Coalition groups means the number of males in a coalition, forming a coalition size. This can be found in line 111 to 120 of the revised manuscript*

- L 118: what specifically about the social group? Group size?

*Throughout the manuscript, we refer ‘social group’ as individuals of the same species living together sharing similar characteristics and interact with one another. Therefore, lions live in social groups of females and males in the prides, and males do form coalitions, whose major role is to ensure survival and protection of the entire pride. Male coalitions can be two individuals, three and above thus having different group size. Group size of male coalition determine the persistence of the lion pride, as they are responsible ensure protection of the females and cubs as observed in line 65 and 68*

- L 181: clarify how key informant interview data were used with respect to your analyses. How many of these interviews were conducted? How were the results used?

*Data were collected from15 key informants found in line 178-180. Data from key informant was recorded in percentage and used for comparison with information obtained in the process of interviewing communities surrounding TME found in line 180 - 181.*

- L 358-360: say how these factors are influenced by location, or remove and start with your second sentence

*The sentence have been removed and the paragraph of the sentence as started with line 326 in the revised manuscript.*

- L 362: be clear that this is trophy hunting. Readers might not distinguish between retaliatory killing and sport hunting as both involve hunting in some form. Be clear in the Introduction and then use consistent terminology throughout.

*From the introduction section in Line 85 to 88 trophy hunting has been elaborated and further explanations in the discussion section Line 315 to 318. Also, retaliation effect to male coalition can be found in the introduction section Line 83 to 86, and later discussed broadly in Line 308 to 312.*

- Starting L 364: it’s not clear why you are bringing in other causes of population decline (e.g., snaring). Be clear in how you are connecting these studies to yours.

*Revision of the discussion section has been done and contradicting text have been removed from line 319 to 320 in the revised manuscript.*

- L 365: state the country locations for these two parks

*Location of the parks cited as examples are in Line 321 to 323, Zimbabwe for Hwange national park and Tanzania for Serengeti and Ngorongoro Conservation Area.*

- L 373: This is not clear. Who did the attacking? And on what? Sub-adult male lions were killed so they did not try to take over prides as they got older? This connection is not clear.

*Contradicting sentences in this section have been removed and the work reorganized and written again*

- L 374: Again, say who did this and then give the citation

*Contradiction in the Line 374 has been removed*

- L 376: please be more specific in what you mean by “human habitats” or use another term

*The line has been removed*

- L 377: whose stability?

*This line has been deleted.*

- L 387: clarify that you are comparing your results to a study in another country

*This is a personal observation made while in TME in line 332 to 334 of the revised manuscript*

- L 389: perhaps use “supporting” instead of “conforming”

*We have changed the word from conforming to supporting in line 337 of the revised manuscript with track changes*

- L 391-392: adjust section headings for proper grammar, here and throughout

*The section heading was adjusted for proper grammar line 338-339 throughout the revised manuscript*

- L 408: “humans”

*Changed to humans line 362 in the revised manuscript*

- L 410 - 441: this is the Discussion, be sure you are discussing (here and elsewhere) what your findings mean, not just stating or re-stating them. Same with the Conclusion. You make general statements about negative impacts, but you don’t discuss what these are or what they mean.

*The discussion part has been elaborated from line 308-379 in the revised manuscript*

- L 431: what determines risk? This needs to be clear from the beginning for your readers to understand your findings. Is this related to the information in SI Table 1?

*Information to elaborate what is defined as risk have been added and elaborated from the introduction towards all section of the paper.*

*Information in Table S1 contains details of the villages surrounding protected area their record of lion killing incidence*

- L 439: which other carnivores? You brought up the idea of “carnivores” previously in the text, but I do not see species other than lions being investigated in your study

*In the result section other carnivore mentioned hyena line 307. Also in the questionnaire survey a list of problematic animals was listed in S2 table. In the discussion section Hyena is mentioned again in line 291 and 364 when compared with lions in level of attack and preference of livestock predated.*

- Figure 1. Is very cluttered and difficult to read. The background colors make it challenging to read the labels. Areas you mention (L 131) are not labeled but others are. Consider what is necessary to label (e.g., location names, pride names given in the text) and what text can be removed. The image is of poor quality to read on enlargement. Not all colors are labeled in the legend. It might be clearer to focus on the prides in Fig 2 as you do and provide the other basic information in Fig 1. You could try substituting the points for polygons for the pride ranges to see if it makes the figure less cluttered.

*Thank you editors for your inputs, we have managed to reduce information in fig 1 map. From line 133 to 138, fig 1 caption has the details of the surveyed villages, and location of hotspot villages were retaliation occurred.*

Table 5: final column header not displaying properly

*The column header has been corrected now found as supporting information (S5 Table).*

- Table 7: fix formatting

*The format has been fixed now found in supporting information (S8 Table)*

- Citation 27, my last name truncated. It should read as Romanach.

*The last name has been corrected.*

Comments addressed from reviewers

Reviewer #1: I did not find any mention on the gender aspect regarding human - lion conflicts

*We thank the reviewer for this useful comment and have addressed this from the introduction section in line 98 of the revised manuscript. We have added information concerning the gender aspect on the revised manuscript. Also, in the GLMM, the variable gender was included but wasn’t significant to the response variables.*

*Moreover, most cultures of communities around TME have labor division according to the age rank and sex. Were young men their main responsibility is to herd livestock and ensure their protection, while women are responsible with home chores. Female gender has little engagement with livestock and most often would only narrate stories once heard regarding livestock or predator attack.*

Reviewer #2: This is an interesting paper addressing an important, yet understudied topic. My "no" response to question 3 is in reference to not seeing the full set of underlying data, from lion population as well as community responses. Further, it seems that much of the analysis on community perception focuses only on a set of the questionnaire data shown in Table 2. Specifically, questions 17-22 seem to provide rich qualitative data but are not addressed in the analysis, results, or discussion sections of the paper. Were the findings from these questions not significant? Or were they incorporated but not described well enough?

*We thank the reviewer for this useful comment and have addressed this in the results, discussion and supporting information section. We have already added the lion population and community responses data on supporting information files (see S10, S11). Also corrections of the questionnaire to reflect what was done during the survey program to some questions was done.*

*Together with that, variables used as predictors (i.e occupation, age class, gender, education level, time period and benefits from conservation were from question seven to twelve. For the analysis of the GLMM we created candidate models for questions 17 to 21, found in supporting information (S5 and S6 Tables). Thereafter significant variable were further tested to get their p values from line 288 to 291, also line 340- 346.*

*For questions 13 to 16, the questions for identifying the major challenge that communities living close to protected areas face, estimating lion population trends their results were incorporated to the discussion section comparing to other studies report conducted in TME and other areas in line 363- 379.*

*From question 13(a –c), the major wildlife challenges community face living close to protected areas were identified, animals causing problem and the seasons were the events occur in line 363 t0 365*

*From question 14 and 15, we identified the protective measure that a household use to safe guard the livestock and examined to what extent the method used has helped to reduce or eliminate livestock predation events in line 350-352 of the revised manuscript.*

*Question 17 and 21 were asked simultaneously to assess community understanding on retaliation effect to lion population in the supporting information (S2 Table) and results were discussed from line 363-379 of the revised manuscript.*

Reviewer #2:There are only two mentions of the factor of human communities blocking migration corridors, leading to negative impacts on the lion populations – Did your QGIS analysis consider an increase in fragmentation and thus an increase in edge habitat, which seems to be the locations where conflict occurs most?

*We thank reviewers comments, analysis from QGIS took into account the presence of permanent settlement of the respondents that leads to reduction of the habitats and blocking migratory corridors for lions. At the periphery of the protected areas where it lies close to communal lands, there is a small buffer zone that has unclear demarcation hence the edge habitat was not clearly defined and wasn’t included in the analysis of the QGIS.*

Reviewer #2: The description of community demographics do not speak to the sex factor, and I do not see a breakdown of your "predictors" in Table 2. Specifically, because you limited your interviews to heads of household, and not knowing enough about the gender norms of leadership in these communities, I wonder if the study group was biased towards males or towards females, and if you gathered insight into how sex might influence lion perception?

*We thank the reviewers’ comments, we have made correction and included in the revised manuscript gender factor in the description of community demographics line 98.*

*From survey of the interview in absence of the family head, the spouse head or even the senior child (aged at least 18 years) of the family was interviewed found in line 177, 180, also a summary of the gender factor in introduction section (Table 1).*

Reviewer #2: I also do not see from the information provided how "benefit" was decided - did a person specify whether they fell into one of the factors within this category?

*We thank reviewers comments and have added information in line 187 to 188 concerning respondents demographics and we have summarized the report of the respondents in line 261 to 265.*

*A person can fall either in one or both of the categories of benefit meaning a respondent can be employed as a tour guide and also report that protected areas have assisted in community development.*

Besides these questions regarding the inclusion of all available data on community perception, there are a few editorial comments mainly in the conclusion section. The sentence that begins on line 394 is not complete. Please check that other grammatical edits are completed.

*We thank editors comments, and have made changes on the caption of line 338 in the revised manuscript.*
